# The Hospital. Nursing Section

**Published:** 1905-04-01

**Authors:** 


### The Hospital.

### Nursing Section.

Contributions for this Section of "The Hospital" should be addressed to the Editor, "The Hospital" Nursing Section, 28 & 29 Southampton Street, Strand, London, W.C.

No. 966.—Vol. XXXVIII. SATURDAY, APRIL 1, 1905.

### Notes on News from the Nursing World.

### THE RIVAL REGISTRATION BILLS.

The Nurses' Registration Bill, of which Mr. Munro-Ferguson has charge, was put down for second reading on Thursday night, but, of course, it was never reached. It has now been placed on the paper for Monday, April 17th, though its sponsors must be aware that it is futile to try to proceed with a private measure on a Government night, and both Thursday and Monday are Government nights. Mr. Claude Hay has brought in the rival measure of the British Nurses' Association, which proposes the compulsory registration of nurses and the voluntary registration of nursing homes! The futility of this proposal must be apparent to all who know anything about the scandals of nursing homes. Of course, it is obvious that as the Select Committee on nursing is to be reappointed, the introduction of these Bills is quite superfluous, even from the point of view of their friends.

### DEATH OF A FORMER MATRON OF NETLEY.

Miss Jane Catherine Shaw Stewart, whose remains, at her own request, were laid to rest at Slinfold, in Sussex, a few days ago, was one of Miss Nightingale's nurses at the military hospitals in the Crimea. On her return to England at the close of the war she was appointed Matron-Superintendent of Netley Hospital, and occupied that position until 1863. Miss Stewart had lived for many years in retirement.

### THE FOREIGN SECRETARY ON NURSING.

Lord Lansdowne presided at the first annual meeting of the Wiltshire Nursing Association, which was held on Saturday at Wilton House, the seat of the Earl and Countess of Pembroke. In the short speech which he delivered on the occasion the Foreign Secretary said that the new departure which the county nursing associations was making was one of the utmost importance. He was sure that all felt that the care of the sick was not a matter to be treated haphazard, but that it was one of the most necessary, and almost one of the most scientific, duties which any human being could perform towards fellow men and women. He knew that people had been moved when they read of the poor soldiers in the Far East lying sick and suffering, with, perhaps, only one nurse to many thousands of sufferers; but he thought that in England we were open to the reproach that the sick and wounded of our daily life had been left too often without proper or skilled care. This was a blot on our social system which he hoped would be removed once and for all by county nursing organisations. We rejoice that with all his obligations as head of the Foreign Office and leader of the House of Lords, Lord Lansdowne finds leisure to show the practical interest which he takes in nursing; and we entirely agree with him respecting the value of county associations, so long as they do not interfere with existing bodies which are doing good work on sound lines, and do not permit anxiety to provide an adequate number of nurses to tempt them to lower the standard of nursing maintained by the Jubilee Institute.

### PRINCESS CHRISTIAN'S NURSES.

The annual report of Princess Christian's Trained Nurses is all that could be desired in respect to the work done. In Windsor, Eton, and Clewer, 673 cases were nursed last year by the district nurses, who paid 14,876 visits. It must be a source of pleasure to them that on the last day of the year the executors of the late Mr. Charles Usher presented a cheque for £200 to the institution "in recognition of the kind treatment of the poor residents in Windsor by her Royal Highness's district nurses." We note that the total income exceeded the total expenditure by nearly £500, but the former includes the earnings of the private nurses. It appears that the sum received from subscriptions on behalf of district work is so small that "a considerable deficit" in this branch has to be met by the profits derived from the work of the private nurses—a position which cannot be considered satisfactory. If the two branches were not combined in one organisation, we feel sure that the numerous well-to-do people in the neighbourhood of Windsor would supply a sufficient amount to provide for the nursing of the sick poor.

### FIRE AT THE NURSES' HOSTEL.

A rather alarming outbreak of fire occurred at the Nurses' Hostel, South Block, on Saturday afternoon, which might have led to very serious results but for the promptitude of one of the nurses who occupied a room at the top of the house. Smelling smoke she immediately investigated and gave the alarm. The entire woodwork of the fireplace in the nurses' sitting-room was burnt out and when discovered the fire had eaten a large hole into the flooring, so that another five or ten minutes might have been fatal. While waiting the coming of the fire brigade, which was considerably delayed, every effort was made, with the help of some passers by, with the result that only a smouldering mass of woodwork remained on their arrival. The South Block is so built that if the front of the building caught fire, escape would be next to impossible for anyone in the back rooms. There are of course a couple of fire buckets on each landing.

### GUY'S NURSES AND THE MILLION SHILLING FUND.

With characteristic enthusiasm the nurses of Guy's Hospital who have taken cards in the interests of the Million Shilling Fund which has been established in order to assist in raising the balance of the amount it was sought to obtain in 1901, are already busily engaged in securing the support of their friends. The spirit of emulation has been aroused, and there will be a keen competition amongst them for the honour of collecting the largest number of shillings. A number of old "Guy's" nurses in different parts of the country have volunteered to help.

### ASSISTANCE OF MEDICAL OFFICERS FOR MIDWIVES.

It will be remembered that in our columns last week the matron of the City of London Lying-in Hospital expressed the opinion that there will be difficulties in carrying out the Midwives Act, because of the awful poverty of many of the women requiring the services of midwives. This is clearly recognised by the Nursing Associations of the four counties of Oxford, Gloucester, Worcester, and Warwick, who have just memorialised the Local Government Board. They have, therefore, asked that directions shall be given whereby the district medical officers of the several Unions shall be required to attend in cases of urgency on the requisition of the midwife whose name is on the roll, and who, for the purpose of such requisition, shall have the same power as an overseer, the medical officer being paid for his services by the guardians according to the scale provided in the event of orders given by a relieving officer. It is also proposed that the case shall be forthwith reported by the medical officer to the relieving officer, who shall bring the matter before the Board of Guardians to determine whether the services of the former shall be treated as medical relief, or as a loan to be repaid in such manner as the guardians shall see fit by the husband whose wife has been attended. We are quite sure that provision of the kind suggested by the Associations will have to be made, if the Act is to fulfil the expectations of its promoters.

### CHELSEA INFIRMARY NURSES AND PENSIONS.

At the invitation of Miss Barton, president of the Chelsea Infirmary Nurses' League, an address was recently delivered to the members on the subject of the Royal National Pension Fund for Nurses by the secretary, Mr. Louis H. M. Dick. His remarks were followed with great interest by the large number of members of the League present.

### AN UNREDEEMED PROMISE AT WEDNESBURY.

There was one unsatisfactory feature in the report of the Wednesbury Nurses' Institute, which was adopted at the annual meeting. The Mayor, in moving its adoption, drew attention to the fact that an item of £14 or £15 from a walking match had not yet been paid in, and urged that steps should be taken to recover the money. It is, happily, very rare that an incident of this kind occurs, and it is in unpleasant contrast to the admirable support given by the workpeople of Wednesbury to the Institute. In 1898 they contributed £39; in 1899, £76; in 1900, £82; in 1901, £86; in 1902, £121; 1903, £143; and in 1904, £165. This steady increase is highly creditable, and it is very encouraging that the greater portion of the income of the organisation is now subscribed by those who directly benefit from it.

### A COMPLIMENT TO BRISTOL.

At the annual meeting of the Bristol District Nurses' Society, Archdeacon Stewart, formerly vicar of All Saints, Knightsbridge, who moved the adoption of the report, said that it was very refreshing, after his London experience, to find that in Bristol the charitable institutions were, as a rule, financially sound; and he congratulated the Bristol Nurses' Society in particular, "upon the good management which enabled them to show a solvent balance sheet." It is true that the balance in hand was only just over £10, but we agree with Archdeacon Stewart that this is a very much better result than many organisations in London are able to achieve. Our columns almost every week also prove that many provincial associations contrast quite as unfavourably with the Bristol Society as some of the London charities. We do not imagine that there is any thing in the air of Bristol to account for the good management which the Archdeacon justly eulogised. The truth is that the solvent condition of the District Nurses' Society in that city is due to the fact that the managers are able and energetic, and that they pursue the right methods in order to obtain support. The pity is that some other committees are wanting in both ability and energy, and neglect the means by which financial success can be attained.

### IRISH NURSES' ASSOCIATION.

The annual meeting of the Irish Nurses' Association was held in Dublin on St. Patrick's Day. The room was crowded, and the President, Miss Huxley, delivered her farewell address. The Association had now, she said, been a year in existence, starting with a balance of £13 handed over from the Nurses' Club, of which the Association had been a development. Subscriptions had amounted to £95, and the expenses had been under £93, so that the Association found itself able to enter upon the second year of its existence in a solvent condition. The lectures given during the winter by various medical men had been much appreciated by the members, as also the social gatherings, and during the summer months cycle rides had been arranged, tea being frequently provided at the homes of friends living outside the city. Miss Huxley congratulated the members on the way in which they had taken their stand during their first year, and then asked them to proceed to the selection of a new President, the Vice-President, owing to press of work, preferring not to serve. Each member wrote the name she proposed on a slip of paper, and Miss Hampson had the greatest number of votes. She was therefore elected President, and Miss Kelly Vice-President.

### GLOUCESTERSHIRE COUNTY ASSOCIATION.

The Committee of the Gloucestershire County Nursing Association have secured the services of Miss Palk as county superintendent. She was until recently chief midwife of the Gloucester District Nursing Society, and has worked, for eleven years, in all branches of nursing in the city of

Gloucester. The appointment may not have the effect of inducing successful organisations like that at Stroud to join the Association, but it will tend to inspire confidence in the new movement generally.

### COTTAGE HOSPITAL NURSES IN NOVA SCOTIA.

It will be learnt with interest that two pupil nurses who have just graduated at the Cottage Hospital, Springhill Mills, Nova Scotia, are the daughters of coal miners working in the Springhill coal pits, and that they left England for North-West America with their respective parents as young children. By great diligence they acquired their present proficiency, and after two years of hard work in the wards, and in district nursing, thay have now received the certificate—or, as it is called in Nova Scotia, the diploma—of a cottage hospital-trained nurse. With respect to the period of training for a cottage hospital, they are ahead of us in Nova Scotia.

### A DEFICIT AT LEICESTER.

It was stated at the annual meeting of the Leicester Institution for Trained Nurses that the expenditure for 1904 exceeded the income by £133, and that this sum had to be made good from the small reserve fund. We regret that there was a deficiency, but it was met in the right way. The institution includes a district branch and a private branch, and instead of the earnings of the private nurses being used to supplement subscriptions to the district branch, the profits on the private branch were distributed in the shape of a bonus to the lady superintendent and the nurses in recognition of their services. The Leicester people, to whom the committee of the institution appeal for sufficient subscriptions in order to enable them to pay their way on the district branch, should clearly understand that they are only asked to assist a work of mercy.

### INDIAN GIRLS AS TRAINED NURSES.

It is proposed to apply to Congress for an appropriation of $20,000 in order to establish a hospital in connection with the Carlisle Indian School. One of the objects in view is to train Indian girls as nurses. It has been found out in the United States that Indian girls can be converted into excellent nurses, partly because they are not troubled with nerves, and partly because they are naturally very quiet. The development of this movement will be watched with general interest.

### A SERIOUS DEFICIENCY IN NORTH LONDON.

The North London Nursing Association, which has just held its annual meeting, has now been established for a quarter of a century, but it cannot be said that the position of the organisation is satisfactory, except in regard to the work of the nurses who last year paid nearly 40,000 visits to 1,642 cases. The financial situation reflects both on the vigilance of the collectors and on the public spirit of the people in the district covered by the operations of the association, as the deficiency is upwards of £200. There is a great falling-off in the sum contributed by the churches and chapels, the amount which in former days was about £200, being now less than £50. Means should be taken by active members of the association to press its needs upon the clergy and ministers of all denominations. Considerable regret was expressed that street processions and collections have been forbidden. We cannot share this regret, and we are convinced that in such a large district other means less open to criticism might be successfully employed to obtain sufficient subscriptions to defray the necessary expenditure.

### MORE NURSES NEEDED AT BELFAST.

The late Mr. Robert Wallace Murray, head of the well-known firm of tobacco manufacturers in Belfast, was in a position to appraise the value of the work of the Belfast Society for Providing Nurses for the Sick Poor, and his will, from which we learn that he left the association £1,000, is a proof of his confidence. The 31st annual meeting of the society has just been held, and the report for 1904, which was adopted, shows that 1,116 cases were attended by the nine nurses, who paid upwards of 35,000 visits, or 1,200 more than in 1903. The expenses were heavier than usual owing to the payment of a night attendant, since the nurses cannot, of course, undertake night as well as day duties. If the society had a larger income it would doubtless be possible to do more in that direction. But, as it is, the expenses last year were £153 in excess of the income, and the staff for day nursing is not adequate. There should, at least, be ten nurses always available for this purpose.

### £20 FOR 225 LECTURES.

A grant of £20 has been made by the Devonport Guardians to the medical officer of the workhouse for extra services in connection with the training of probationers during the past five years. In this period he has given 225 lectures to the nurses, and as the delivery of lectures is not included in the ordinary duties of his position, we cannot congratulate the guardians upon their liberality. Even the paltry sum of £4 a year was opposed by some members of the board, though we cannot but think that, if the ratepayers desire the probationers in the Poor-law Infirmary at Devonport to be trained in such a manner as to reflect credit on the institution, they would not grudge a grant of £10 a year to the medical officer for his share in the work.

### LOWESTOFT PROVIDENT NURSING ASSOCIATION.

The Mayor of Lowestoft in moving the adoption of the report of the Provident Nursing Association at the annual meeting, warmly eulogised the work of the district nurse who paid upwards of 3,000 visits last year. He supported a recommendation in the report that the Kirkley Nursing Association should be asked to amalgamate with the organisation, and it was agreed to. The financial position is very satisfactory, the receipts being £236 as against expenditure £208. With regard to the inclusion of Kirkley, however, the rector of Lowestoft urged that £79, the sum contributed by 100 subscribers, ought to be quite doubled.

### ST. MARY'S DAY AT ST. MARY'S HOSPITAL.

There was a special service in the chapel at St. Mary's Hospital, Paddington, on Saturday, the Feast of the Annuciation. The music was of a special character. "Oh, for the wings of a Dove" was sung by the Home Sister, "Calvary," by Nurse Thorpe, and two sacred part-songs by some members of the Students' Choral Society.

### The Nursing Outlook.

"From magnanimity, all fear above;
From nobler recompense, above applause,
Which owes to man's short outlook all its charm."

### THE NURSING MIRROR.

The success of a newspaper can best be tested by its circulation, its usefulness, and its reputation. The Hospital has good reason to be satisfied when so tested, as the results of 19 years' publication abundantly show. These years have been fruitful of much which has contributed to make the modern hospital the wonder and pride of the twentieth century. In no portion of the hospital field have the developments attained exceeded those reached in the department of nursing. Twenty years ago nursing as it now exists was in its infancy, and the number of trained nurses compared with that of to-day was relatively insignificant. So popular has the nurse become in the homes of the people that in addition to the army of certificated nurses who now supply the hospitals and upper and middle class homes, the district and parish nurses of various grades constitute an army of devoted women, who spend their lives in ministering to the needs of the humblest subjects of the King. We have striven always to meet the increasing claims and practical needs of nurses of all grades in these columns. From the very outset there has existed a depth of sympathy between the editor and his readers, which has united them from the first and has been fruitful in results for the good of patients, of nurses, and all who are interested in both alike. It is well sometimes to recall and to recognise a long and pleasant unity of this kind, and to-day there is a special reason for such a step.

The Hospital has met and on the whole supplied successfully a need, which was recognised by all classes of hospital workers and especially by the nurses, their teachers and employers. Amongst the 80,000 nurses or more now working in the British Empire are a great number of highly intelligent and educated women, whose earnings are reasonably large, and whose interests are wide enough to include the whole field of hospital work—that is everything of interest, everything which affects the welfare of the patient, the advancement of sound hospital administration, and the whole field of nursing. To all such The Hospital has come to be well-nigh indispensable, and no greater pleasure has been granted to us in the course of a long and busy life than the many expressions of goodwill and appreciation which have filled the editorial letter-box each Christmas and New Year.

It has been represented to us by a great many nurses that, in addition to those whose needs The Hospital has supplied, there are a large number who desire to have placed at their disposal a weekly newspaper, purchasable for one penny. It is therefore our pleasure to announce that on April 8th we shall publish a useful and practical weekly journal for nurses at this price entitled The Nursing Mirror, which will deal exclusively with nursing in all its branches. It will contain a section for post-graduate nurses, lectures and papers upon subjects of special interest to probationers and nurses generally, information concerning all nursing movements and developments at home and abroad, general and personal news, correspondence, notes and queries, travel notes, the book of the week, an account of the latest appliances and novelties, and everything, indeed, which is calculated to help, to interest, or to instruct all who are engaged in the field of nursing. The Nursing Mirror will in fact be identical with the present Nursing Section, and will contain 32 pages of the same size as those of The Hospital, with illustrations and diagrams in addition as opportunity may offer. We hope in this way to embrace every nursing interest, and to aid many thousands of readers who, for various reasons, may not at present subscribe to The Hospital. As the demand is likely to be very great those nurses who desire to have a copy should at once order The Nursing Mirror from the nearest bookstall or newsagent. In no surer way can disappointment be avoided.

We have also determined to still further meet the wishes of our readers by setting aside a room in The Hospital Building, Southampton Street, Strand, for business appointments where advertisers can have interviews on business by arrangement through the manager. This room will, we believe, prove helpful to nurses especially who come to London from the country for a few days, and they can have their letters addressed of The Hospital Building, where an opportunity will be given to nurses to see the latest nursing books, publications, and literature. Advertisers, too, will be granted the privilege of having their advertisements inserted in "The Nursing Mirror," as well as in the nursing section of The Hospital, without any extra charge. We hope that every reader and advertiser will make these facts known to their friends, and that the result may prove in every way satisfactory to our readers and ourselves.

The Hospital has been a rallying point for nurses from the outset. It has formed the link between the nurses who have gone out to labour in the more distant parts of the Empire, their alma mater, and their friends at home. In this way, thanks to the co-operation of our readers in all parts of the Empire, we have been enabled to supply information and to excite an interest in nursing work in every centre however distant where good work is being done. The more our readers will co-operate by sending to us reports, information, and items of interest, the better shall we be able to fulfil our mission as a newspaper for nurses. In thanking our readers for the generous support they have invariably extended to The Hospital we venture to express a hope that this further effort to supply the needs of every nurse will stimulate an increasing number of nurses, to enter into a regular correspondence with the Editor, who is always glad to supply information as well as to receive it.

### Lectures on the Nursing of Sick Children.

By James Burnet, M.A., M.B., M.R.C.P.Edin., Registrar, Royal Hospital for Sick Children; Clinical Tutor, Extramural Wards, Royal Infirmary; and Physician to the Marshall Street Dispensary, Edinburgh.

### III.—BATHS. WHEN AND HOW TO GIVE THEM.

Although the attending physician will, to a certain extent, give orders regarding the bathing of his patient, he usually trusts a good deal to the nurse's intelligence to see that this important matter is carried out.

The bathing of a sick child is a matter requiring considerable knowledge and skill on the part of the nurse. Even among the educated classes there is usually considerable difficulty in persuading the parents that their child, though ill, nevertheless requires his skin kept in a hygienic condition. In not a few cases the nurse will get herself into trouble if she attempts to bath the child on her own responsibility. She should, therefore, always refer the matter to the medical attendant in presence of the parents, with a view to obtaining his sanction first in every case.

When the child is too ill to have his usual bath he may be sponged over while lying in bed or in the nurse's arms. In such cases the lower limbs and abdomen should be first sponged over with tepid water and then thoroughly dried. Thereafter the chest, and finally the face and upper limbs may be attended to. The process should be carried out, if possible, in front of the fire, and the patient should immediately afterwards be put to bed between blankets which have been previously warmed.

If the condition of the patient is such as to admit of a hot bath this should be given preferably at bed time, as the bath has a tendency to promote sleep. The temperature of the water should be between 100° and 105° F. Care must be taken against draughts, and it is often advisable to have a screen round the bath while the child is in it. The whole process should not occupy more than three, or at most five, minutes; and when the child has been bathed the skin should be rapidly but thoroughly dried. The patient is then to be put to bed and given a warm drink. If the child looks blue and cold the bath must not be repeated, but tepid sponging should as a rule be substituted.

Baths, however, may be given for other purposes besides that of bodily cleanliness. Thus, in febrile conditions, cold sponging may be of the greatest possible service in reducing the temperature. Not infrequently the actual cold bath is ordered to be given. In this case the nurse places the patient first in a warm bath, the temperature of which should never be less than 90° to 95° F. To this ice or cold water may be very slowly added until the bath thermometer registers 70° or even 65° F. The whole procedure should not occupy more than five minutes, and a few sips of hot milk should be given while the child is in the bath. If the pulse become feeble and the temperature fall to normal the patient must be taken out of the bath at once, and a few drops of brandy-and-water administered. In all cases the child should be got back to bed as quickly as possible.

A hot mustard bath is often useful in cases of collapse or of great exhaustion, such as is often seen in severe diarrhœa. The temperature of the water should be not less than 105° F. To the bath mustard paste should be added, in the proportion of two table-spoonfuls to the gallon. The paste is made by mixing mustard with cold water in the usual way. It is of no use whatever to simply throw mustard flour into the water, as the mustard will run into lumps and not mix with the water in the bath at all. Such a bath is very useful in cases of threatening heart failure, so often met with in the pneumonia of young children.

The cold douche is very valuable as a means of treatment. It acts as a tonic to the nervous system. When the cold douche is ordered the nurse is expected to know how to give it. Unfortunately few nurses seem to understand its use. Always remember its action is purely as a tonic. It is often prescribed in cases of rickets, as well as for nervous children and those who are the subjects of chorea. It is beneficial in those who are debilitated and anæmic, and its use often improves the appetite and general condition of the child. It may be given immediately after the ordinary morning bath, but as a rule it is advisable to give it separately. The child should stand or kneel in a bath containing a few inches of warm water, and the nurse should take a large jug containing water from which the chill has just been taken off. This is to be poured upon the child's back. Two, or at most three, jugfuls will suffice, and then the child should be well rubbed down for five or ten minutes before the fire with a coarse bath towel. He should not be allowed to play about half-dried, as is so often the case when the nurse is at all careless.

Alkaline baths are sometimes ordered for skin affections. Two tablespoonfuls of carbonate of soda should be added to a bath containing six gallons of water at a temperature of 100° F. The child should remain in the bath for at least ten minutes. Salt-water baths may be prepared by adding common salt (not necessarily sea-salt) to water at a temperature of 80° to 85° F.

The hot-air bath is one which the nurse may be called upon to give in cases of kidney disease. For this somewhat elaborate preparations are necessary. The bed is made ready by placing a piece of waterproof sheeting covered over with a blanket below the patient. Next a cradle is put over the child's body and limbs, and the cradle in turn is enclosed in a waterproof covering. Over this several blankets are placed which must be firmly secured by fixing their ends under the mattress. At the end of the cradle is a wooden frame with a hole in the centre through which the funnel of the hot-air apparatus is passed. As soon as the child begins to perspire freely the funnel is withdrawn, and the hole is covered over by a circular disc made to fit accurately. The cradle is then removed by pulling it downwards, and the patient completely surrounded by the blankets which are left in position for an hour or two. He is then placed between fresh warm blankets and given warm drinks, and if necessary other stimulants.

Some General Points to be Attended to.

When a sick child is cross and fretful, refusing to sleep, and generally causing the nurse no end of trouble, a hot bath will often produce a very soothing effect. A baby who has been crying all day and exceedingly irritable will often be sent to sleep by giving him a hot bath to which a small amount of mustard has been added. If a child turns cold and blue, cries feebly, and shows signs of shivering, the nurse should at once remove him from the water. dry thoroughly, and put him to bed with all possible speed. Such a child will need to be wrapped well up in blankets, and have two or three hot-water bottles placed round him. A glass of hot milk or a small cupful of beef-tea should be given him to drink, and if these measures do not suffice, a few drops of whisky or brandy and water may be administered. In such cases it is well on future occasions merely to sponge the child as he lies in bed or in the nurse's arms before the fire.

In the case of babies great care must be taken in drying the patient. If too coarse a towel is used the skin may easily become abraded. So too if the skin is not thoroughly dried, it will become fiery and irritable. As a rule it is well to use a powder-puff in every case. The best material for general use is one containing equal parts of rice and starch-powder. These require to be very carefully triturated, as otherwise the powder is apt to cake upon the skin. Special attention should be given to the various folds in the skin, such as the groins, armpits, and neck. Fuller's earth should never be used, as it is not nearly fine enough for this purpose. As a last point, we would say that cold baths should be given in the morning and hot baths at night unless otherwise ordered by the physician.

### The Nursing of Diphtheria in a Private House.

### EXAMINATION QUESTIONS FOR NURSES.

The question was as follows:—If called upon to nurse a case of diphtheria in a house where means are very limited, and appliances such as are used in hospitals not to be obtained, what arran ements should ou make?

### First Prize.

I should choose a room at the top of the house, and one adjoining if obtainable, removing unnecessary articles of furniture, carpets and curtains, having a plain iron bedstead, preferring one with a wire mattress and having only a wool mattress upon it. It should stand away from the walls, to allow of a free current of air all round. I should nail a sheet, wrung out of disinfectant, over the outside of the door, reaching to the floor, kept constantly wet. A table placed outside to receive anything required for patient, also food, which could be transferred to utensils used by patient.

I should use the adjoining room in which to keep all articles used by patient.

I should have free ventilation, keeping the temperature at 60° F. for an ordinary case, and 65° F. to 70° F. where tracheotomy had been performed. Having a small fire constantly in the room, I should keep the air moist by means of a kettle boiling upon it, supplying this from another boiled in the adjoining room.

If a steam tent were ordered, I should arrange it either by means of a clothes-horse placed round the bed, over which I should place sheets forming a tent, or by fixing four long pieces of wood, such as broom handles, one at each corner of the bedstead, tying stout cord at the upper ends from one to the other, over which I should arrange sheets to form a canopy covering in three sides and the top. I should conduct the steam from the kettle by means of stout brown paper made to form a hollow tube, fixing one end to the spout of the kettle, the other reaching into the tent. For spraying the throat, an ordinary scent spray will do, for painting it a small mop could be made of old rag, fastened to the end of a stick and afterwards burned. The tongue could be depressed with the handle of a tooth brush. An old unused teapot or jug with a cloth placed round the top, makes a good inhaler. A jar with a lid could be used as an expectorator, containing disinfectant or else lined with paper, which could be lifted out frequently and burnt with its contents, and the jar boiled. Old pieces of rag should be used to wipe away discharges from nose and mouth and should be burnt at once. Any linen soiled in the same way could be carried into the next room and be boiled for a few minutes in an old saucepan or fish kettle kept for the purpose. A pail or bath outside the room door containing disinfectant could receive this linen afterwards for washing. All utensils used by patient washed and kept in disinfectant and boiled. Hot flat irons, or bricks heated in oven, and covered with flannel make good substitutes for hot water bottles. Patient's room cleaned and dusted by means of old cloths wrung out of disinfectant and afterwards burnt.

"Central."

### Second Prize.

The case must be isolated at once in a large (if possible) well-ventilated room on the top floor, where no draughts are permitted to enter. Everything must be removed that is not wanted for immediate use. A fire must be kept burning and the room kept at an even temperature of 65° F. Not forgetting the case is a very infectious one and from the beginning must be treated as such and every precaution taken to ensure and guard against the spread of infection. The case is most likely to be a child; he must have a hot bath in the room (an ordinary washing-tub will answer well) and put to bed. The temperature must be taken and the bowels opened with castor oil (enemata preferable, if possible). The urine must be measured, as the quantity of this often indicates much. In the meantime send for urgent necessaries and report to the medical attendant the conditions of the patient. All these orders can be given through the door, which must be kept closed and a damp sheet hung up to cover it inside, which afterwards can be disinfected when the antiseptic arrives. I should give orders to have a second sheet well moistened with the antiseptic solution and hung up so as to cover the whole door outside before I ventured to open it. If the throat is painful hot fomentations must be applied to the neck frequently, or even hot flannel, and hot drinks of milk or anything suitable that is obtainable.

The air of the room must be kept moist. Place a kettle on the fire and see that it is kept boiling. If this is insufficient, a red-hot brick must be placed in a shallow pan containing boiling water. This, with the help of the kettle, will soon impregnate the room with the moisture required. If a tent is necessary it should be a half one, made with a screen and a sheet over the top, or a linen horse, and a sheet arranged round the sides as well as the top, and fastened with pins. Then a second brick will be required, to be used alternately with the first. The pan must be placed on a chair beside the bed and a kind of a hood made (with the waste sheet that is hanging over), so as to direct the vapour into the tent, and the patient will get the full benefit of it. Have a supply of old linen or rags to wipe away discharges, and burn immediately after use. Nothing whatever must be allowed to leave the room without being properly disinfected. Have tracheotomy outfit at hand properly sterilised and ready for immediate use in case of emergency, with brandy and a hypodermic syringe. After the doctor's visit the nurse would, of course, carry out his instructions.

"Lanoitan."

THE PRIZE WINNERS.

The standard of merit reached by the papers this month is not so high as that attained in January. Diphtheria is a difficult disease to nurse in circumstances where poverty is present and where therefore suitable appliances cannot be obtained. The question was put with a view to drawing out the ingenuity of the nurse as exemplified in her ability to make the most of the meagre resources at her command, not, as many candidates seem to think, to test their knowledge of the symptoms of the disease. "Central" again takes the first prize. She deserves congratulation as she has been successful three times in succession. By our rules she will not be eligible for a prize again until next December, but she can compete and be awarded honourable mention. She mentions one small but important item in her arrangements, not noticed by many of the other competitors, namely, that of filling the kettle used for steaming purposes from another boiling one. Some thoughtless nurses would fill the steamer with cold water and so check the flow of steam for some time. "Lanoitan" gains the second prize with a good and practical paper, but says nothing of how he should arrange an amateur expectorating cup.

Both papers are good, but they leave something to be desired.

Neither the prize-winners nor other competitors differentiate sufficiently between the infection of diphtheria and that of other diseases. Remember that the infection of the disease under discussion is mainly present in the discharges from the throat and nose. The nurse is the person chiefly in danger, because other members of the family would not be allowed in the sick-room. Now very few nurses have said anything about caution in wiping their faces or hands if the patient has coughed small portions of discharge over them. It is a misfortune that constantly happens; never neglect to wipe the place at once with some disinfectant. Never take food without washing hands and lips and gargling the throat thoroughly. For the same reason burn all remnants of food in the sick-room, that no one may be tempted to take the delicacies that come from the patient.

HONOURABLE MENTION.

This is gained by "Rhoda," "Nurse Marie," and "Auld Reekie." "Rhoda" gives no proper name; will she kindl send name and address that she may receive her card.

QUESTION FOR MARCH REPEATED.

If permitted by the doctor to choose your own means and hours, in what way would you prepare a patient for operation with regard to clearing the bowels.

THE EXAMINER.

RULES.

The competition is open to all. Answers must not exceed 500 words, and must be written on one side of the paper only, without divisions, head lines, or marginal notes. The pseudonym, as well as the proper name and address, must be written on the *same* paper, and *not on a separate sheet*. Papers may be sent in for 15 days only from the day of the publication of the question. All illustrations strictly prohibited. Failure to comply with these rules will disqualify the candidate for competition. Prizes will be awarded for the best two answers. Papers to be sent to "The Editor," with "Examination" written on the left-hand corner of the envelope.

In addition to two prizes honourable mention cards will be awarded to those who have sent in exceptionally good papers.

N.B.—The decision of the Examiner is final, and no correspondence on the subject can be entertained.

Any competitor having gained three prizes within the current year shall be disqualified from taking another until 12 months shall have expired since the first prize was gained.

### The Central Midwives Board.

An ordinary meeting of the Central Midwives Board was held at the Board-room, on Thursday last week. There were present: Dr. Champneys, in the chair; Dr. Cullingworth, Dr. Ward Cousins, Mrs. Latter, Miss R. Paget, Sir William Sinclair, and Mr. Parker Young.

A NEW MEMBER.

The correspondence included notifications from the Royal Colleges of Physicians and Surgeons, and the Society of Apothecaries of the re-election of their respective representatives, Dr. Champneys, Dr. Ward Cousins, and Mr. Parker Young to serve upon the board for the year ensuing April 1st next. A letter from the Incorporated Midwives Institute gave notice of the election of Dr. W. R. Dakin, President of the Obstetrical Society of London, as their representative on the board for the ensuing year, in place of Dr. Cullingworth. At this point a vote of thanks to Dr. Cullingworth for his valuable services to the board since its work commenced, was carried "with acclamation," the chairman remarking that his loss to the board could hardly be repaired, remembering the amount of tedious, onerous work which Dr. Cullingworth had ungrudgingly undertaken. Miss Paget added that the warmest thanks of midwives were due to Dr. Cullingworth for the consistent fairness of his attitude towards them.

AN IRISH QUESTION.

Letters were read from Guardians of the Carlisle Union, and the West Riding County Council. It was agreed to reply to a letter from Dr. J. McLeish, asking the board to reconsider its decision not to recognise the certificate of the Belfast Union Maternity Hospital under Section 2 of the Act, on the ground that it would be an injury to a number of midwives, including Queen's nurses in Ireland, that the Board saw no reason to make any change. It was pointed out that the Act did not apply to Ireland, and Miss Paget stated that the Queen's Institute had given their nurses in Ireland the opportunity of coming over for the last L.O.S. examinations, so that there was no question of hardship so far as they were concerned. Letters were read from Liverpool asking that an examination centre might be established in that city, and it was agreed to reply that the present arrangements as to provincial centres was a provisional one.

THE RULES IN WELSH.

Some discussion took place with regard to a request from a Welsh Urban District Council that the Board's rules should be translated into Welsh. Sir W. Sinclair contended that such a course was unnecessary, as he did not believe that Welsh midwives could not speak English. If they did not read English they probably could not read Welsh. Mr. Parker Young thought the request a reasonable one, and he also considered that if such a translation were desirable it should be an official one. It was ultimately decided to ask the County Councils Association to give their opinion as to the amount of demand for such a translation.

A letter from Dr. Sergeant, Medical Officer of Health, Lancashire, was next dealt with, asking the Board's opinion as to the operation of Rules E. 17 (c) I. (5) and E. 7. The Board were of opinion that with reference to the first, the attention of any midwife unacquainted with the use of the clinical thermometer should be called to the rule, and that she should be instructed forthwith. The Board agreed with regard to the second question that any midwife refusing to use the prescribed antiseptics after being in contact with infection would be guilty of contravening the rules of the Board. Applications for certificates were passed, the total number of midwives now on the roll being 18,231. Of these

6,174 hold the certificate of the Obstetrical Society of London, and 10,162 are women in "*bonâ fide* practice," the remainder holding other certificates approved by the Board.

The report of the Standing Committee was then taken, and the various recommendations made adopted. The following institutions were approved as training schools:—The Belfast Maternity Hospital; the New Hospital for Women. Approved as teachers under Rule C.I. (3):—Ethel F. Lamport, L.S.A., M.D.Brx.; James Hayward Willett, M.B., Ch.B. (Vict. and Liverpool); Hugh E. Wingfield, M.D., B.C.Cantab.; Charles R. Wood, M.D., B.S.Durh. The following certified midwives were approved under Rule C.I. (2):—Helena Perry Margaret M. Baxter, Grace O'Flaherty, Alice S. Gregory, Hilda Marsland, Lelia Parnell.

#### The Appointment of Inspector.

The following resolution was moved by Sir William Sinclair:—"That the advertisement issued by the board in pursuance of a resolution of January 26th be withdrawn, and the question of the appointment of an inspector be further considered by the board." Sir William Sinclair gave at some length his reasons for moving this resolution, based upon the view that many medical men throughout the country did not like the Act, and that the appointment of an inspector was "flouting the medical profession." Really efficient inspection of any institution would be by the local medical officer of health. He also objected to the board being in such a hurry in the matter, and said that he had never seen the notice on the agenda. The chairman pointed out that the appointment of the inspector had already been made, and it was further shown that the matter had been duly on the agenda and due notice given, but no intimation had been received from Sir W. Sinclair that he wished the question postponed. The resolution not being seconded, the discussion was not continued.

### Everybody's Opinion.

[Correspondence on all subjects is invited, but we cannot in any way be responsible for the opinions expressed by our correspondents. No communication can be entertained if the name and address of the correspondent are not given as a guarantee of good faith, but not necessarily for publication. All correspondents should write on one side of the paper only.]

### SIR JULIAN GOLDSMID'S HOME OF REST FOR NURSES.

Mrs. McIntyre writes:—Will you allow me through the columns of your valuable paper to thank the very many nurses who have written to express their regret at my resignation as matron of "Sir Julian Goldsmid's Home of Rest for Nurses," Brighton, and also to express my gratitude for their kind sympathy and good wishes for my future. I am unable at present to reply to each personally.

### REGISTRATION IN AUSTRALIA.

Miss E. Glover, St. Ives, East Melbourne, writes: The remark made at one of the meetings of the R. V. I. N. Association—"that in spite of registration the competition of untrained nurses in the Colony is increasing" is not correct. As Honorary Lady Superintendent of one of the largest private nurses' homes in Melbourne I can prove to the contrary. There are, of course, many untrained midwifery nurses employed, and will be for some years, the benefit of registration will be felt more in the future, than at the present time. The untrained nurse and the Gamp are slowly, but surely, passing away, and those that are left have much more difficulty in obtaining work than formerly. New Zealand refuses to recognise the training in New South Wales because they have no central board of examiners; Victoria may before long follow this lead, and exclude not only New South Wales nurses, but also those from other countries. Your interest in all that pertains to nursing matters is so great that it is difficult to understand the position you take with regard to State registration.

### MR. SYDNEY HOLLAND AND REGISTRATION.

Mr. Sydney Holland writes from the London Hospital, E.: In reply to "Onlooker," who writes as a trained nurse, I wish, if she has the time, she would communicate with me direct, as it is difficult to answer all her questions within the space available. She writes that she cannot understand the side I take because she has always heard that I "have been a good friend to the trained nurse." Of a truth that is the very reason why I object to a scheme which will, in my judgment, do harm to all good nurses, and bolster up the indifferent and even the bad nurses. How, "Onlooker," can this register provide for the "after years"? You pass an examination; you get to be a "Registered Nurse." How have you got anything of more value than your training-school certificate? Being on the register does not protect the public from "a short-sighted, silly, or even worse" nurse; on the contrary, it would enable such a nurse to get employment because the deluded public would think they need make no further inquiry if a woman is "on the Register." "Onlooker," I think, confuses the registration of nurses with a directory. I would welcome a good directory which, in as large print as possible, stated, "No nurse by being in this directory is guaranteed to be a good nurse. All that is guaranteed is that the entries as to where the nurses were trained are accurate." "Onlooker" has taken out of its context what I wrote about the need of registration for midwives as being applicable to one special technical art, and has, of course unintentionally, misrepresented my meaning. I quite admit "Onlooker" that a chairman may also get "shortsighted, silly, or even worse," which is an excellent reason for your opposing any State registration of chairmen. But, there is this great difference. A chairman can be turned out of his post by those over whom he presides when he becomes silly, and long before he becomes "even worse." A State registered nurse, similarly afflicted, would and must "remain on the register" to the detriment of every good nurse in the profession, because she could not be turned off except for some act almost of criminality.

∴ We are glad to note that Mr. Sydney Holland now approves the publication of a Nursing Directory on the lines we advocated so long ago as January 1895. A small Editorial Committee of Lecturers to nurses and hospital managers have periodically issued such a book as Mr. Holland refers to, entitled "Burdett's Official Nursing Directory" since 1898, which might be taken over by the nurse-training schools.—Ed. The Hospital.

### THE GERMAN TREATMENT OF APPENDICITIS.

"L. F. P." writes:—I have recently been staying in a tiny village in Switzerland, among magnificent scenery. The place is very healthy, but, strangely enough, during my stay of three weeks, three medical cases, and four accidents came under my care. Two days after I arrived an English lady who had only just come with her husband was taken seriously ill with appendicitis. A Berlin doctor was staying in the village and his treatment was different to any I have come across in 14 years' experience; it may be interesting to others. There was considerable distension and very great pain; for two days during the first week it was a question whether the patient would get better. Compresses of spirit, brandy, whiskey, or methylated were ordered over the whole abdomen, with an ice-bag over the region of the appendix; milk, milk and rice-soup, egg beaten up, 3 oz. of brandy were given by mouth, tinct. opium ♏ x doses and mist. bismuthi. The pain and distension gradually subsided, and by the eighth day had entirely disappeared, but the bowels had not been open. On the eighth day a large oil injection was ordered, there was no Higginson syringe to be had, but there was an irrigator which was used, nearly the whole of the oil ℥xv was retained till the next day, when the patient passed two very large motions without much effort. She made a good recovery, the bowels were opened every day after this, fresh and stewed fruit being plentifully given. The doctor told me he had had 100 cases of appendicitis, had never lost a case, and never operated unless he suspected pus or gangrene. This was his routine treatment and he allowed the patient to go on to the 14th day without the bowels being relieved if he thought it necessary, he said he had had cases of recurrence, but he used the same treatment and always found a second attack milder than the first. He could not speak English and very little French. I cannot speak German, but we were fortunate in getting an interpreter most of the time. When not available the conversation was carried on in a mixture of English, French, German, and a plentiful sprinkling of Latin, which last was most useful. There was no ice-bag to be had, and I used a good many sponge-bags which are not satisfactory. I should like to know if other nurses have come across this treatment for appendicitis.

### Appointments.

[No charge is made for announcements under this head, and we are always glad to receive, and publish, appointments. The information, to insure accuracy, should be sent from the nurses themselves, and we cannot undertake to correct official announcements which may happen to be inaccurate. It is essential that in all cases the school of training should be given.]

Chesterfield Infirmary.—Miss S. E. McCurdy has been appointed superintendent nurse. She was trained at Birkenhead Infirmary, where she has since been sister. She has also been night sister at Chesterfield Infirmary. She holds the certificate of the Central Midwives Board.

Cottage Hospital, Pembroke.—Miss Grace B. E. Morgan has been appointed matron. She was trained at the Royal Hospital, Portsmouth, and has since been sister at the North Riding Infirmary, Middlesbrough, and taken holiday duty as night sister at Gravesend Hospital, and as Matron at Monmouth Hospital. She has also done private and district nursing.

Eastville and Stapleton Infirmary.—Miss Helen Jane Dobbins has been appointed charge nurse. She was trained at Boston Hospital, and has since been nurse at Hartlepool Union, West Derby Union, and the Parochial Hospital, Dundee.

Fever Hospital, Birkenhead.—Miss Agnes Routledge has been appointed charge nurse. She was trained at the Clayton Hospital, Wakefield, and the Monsall Fever Hospital, Manchester. She has since been charge nurse at the Women's and Children's Hospital, Leeds.

Hertford and Ware Joint Hospital.—Miss F. Phillip, and Miss E. Allen have been appointed charge nurses. Miss Phillips was trained at the St. William's Hospital, Rochester where she was afterwards charge nurse. She has since been at the Seamen's Hospital, Greenwich, and has also done private nursing at Hendon. Miss Allen was trained at St. William's Hospital, Rochester, where she has since been charge nurse.

High Peak Isolation Hospital, Chinley.—Miss Millicent Oliver has been appointed staff nurse. She was trained at the High Peak Isolation Hospital, Chinley.

Infectious Diseases Hospital, Ashton-in-Makerfield.—Miss Louisa B. Freeman has been appointed matron. She was trained at Salop Infirmary, Shrewsbury, and the Isolation Hospital attached to Chester Infirmary. She was four years at the Acland Homes, Oxford; has been staff nurse at King's College Hospital, London; in charge of a large temporary hospital at Worthing during the epidemic of enteric fever in 1893; matron at Kidderminster Borough Hospital, and matron at the City of Gloucester Isolation Hospital.

Medical Mission, Labrador.—Miss Florence Hilda Bailey has been appointed nurse. She was trained at Bethnal Green Infirmary, London, and has since been staff nurse at the Cottage Hospital, Cowes, and charge nurse at Horton Infirmary, Banbury.

Royal Asylum, Glasgow.—Miss Emily S. Lett has beea appointed assistant matron. She was trained at Sir Patrick Dun's Hospital, Dublin, and has since been staff nurse at a Dublin surgical home, sister at Darlington Fever Hospital, and assistant matron at Sir Patrick Dun's Hospital, Dublin.

Seacroft Hospital, Leeds.—Miss B. J. Goodall has been appointed sister. She was trained at St. Thomas's Hospital, London, and has since been sister, men's medical and surgical wards, at the Royal Portsmouth Hospital.

Stamford, Rutland, and General Infirmary.—Miss Alice Mary Browne has been appointed matron. She was trained at the London Hospital, where she was subsequently sister. She has since been sister at the Seamen's Hospital, Greenwich, lady superintendent and secretary at the Mothers' Seaside Convalescent Home, Herne Bay, and assistant matron at the General Hospital, Birmingham.

### Presentations.

Lodge Moor Hospital, Sheffield.—Miss C. M. Duffy was the recipient of some very handsome presents on leaving Lodge Moor Hospital, Sheffield, to take up her new duties as matron of the Leicester Borough Hospital. By the medical superintendent, medical officers, the matron, and Miss Robinson, she was presented with a silver "Queen Anne" tea service, including teapot, sugar bowl and tongs, cream jug, china tea servers and oak tray. The nursing staff gave her a silver-mounted dressing-case, and the domestic staff and porters a silver tea-kettle and stand. There were several other pretty and useful gifts.

Leeds Union Infirmary.—A presentation was made to Miss Mary D'Evelyn, assistant matron of the Leeds Union Infirmary, on Thursday, last week, of a pair of chased silver bon-bon dishes from the nursing and medical staff. Miss D'Evelyn is leaving the infirmary to be married. The nurses after the presentation had a fancy-dress dance in their recreation-room.

Great Yarmouth Workhouse Infirmary.—On Tuesday, 28th, Dr. H. Collier, Medical Officer of Great Yarmouth Workhouse Infirmary, was presented with a gold card case engraved with his monogram and crest; also a silver and ivory paper knife from the nurses, as a mark of their esteem and goodwill on his retirement.

### To Nurses.

We invite contributions from any of our readers, and shall be glad to pay for "Notes on News from the Nursing World," or for articles describing nursing experiences at home or abroad dealing with any nursing question from an original point of view according to length. The minimum payment is 5s. Contributions on topical subjects are specially welcome. Notices of appointments, letters, entertainments, presentations, and deaths are not paid for, but we are always glad to receive them. All rejected manuscripts are returned in due course, and all payments for manuscripts used are made as early as possible after the beginning of each quaater.

### Wants and Workers.

Nurse Stacey, Chivelston, Wimbledon Common, S.W., has copies of The Hospital from March 12, 1904, to the present date, which, as she is leaving Wimbledon, she will be glad to send to anyone who is willing to pay the postage.

### The Nurses' Bookshelf.

Elements of Anatomy and Physiology Especially Adapted for Nurses. By W. Bernard Secretan, M.B.(Lond.), F.R.C.S.(Eng.), L.R.P. (London: The Scientific Press, Limited, 28 and 29 Southampton Street, Strand, W.C. 1905. 26 Illustrations. 70 pages. Price 2s. net.)

At first sight it would appear that there are already too many text-books of a similar character to the above on the market to give any assured probability of success to another, yet, a second glance at this useful little volume will compel a careful reading of the whole, with the final conclusion that there is ample room on the nurse's bookshelf for this most carefully thought-out and clearly-worded little manual. The utmost brevity, consistent with extreme lucidity, marks the style from beginning to end, and although nothing of importance to the learner is omitted, there is a refreshing absence of those bypaths of miscellaneous instruction which appear in many such text-books, and which, instead of informing, often only succeed in puzzling those for whose benefit they are written. The Course of the Circulation is treated in a particularly clear manner, as also is the physiology of respiration, both of which processes are often as shoals and quicksands to a probationer attending her first set of hospital lectures. The book is well paragraphed and indexed, and the diagrams clearly and carefully drawn. Dr. Secretan is evidently thoroughly acquainted both with the subjects he teaches and the best manner of imparting his information.

Practical Nursing. By Isla Stewart, Matron of St. Bartholomew's Hospital. (London: Herbert E. Cuff, M.D., F.R.C.S., Medical Superintendent, North-Eastern Fever Hospital, Tottenham. William Blackwood and Son. A new edition. 5s. net.

The excellent production, accurately styled "Practical Nursing," by Miss Isla Stewart and Dr. Herbert Cuff has been so widely appreciated that the publishers have just issued a new edition in one volume. At the time the original work made its appearance we dealt with it at some length, and we need therefore only record our gratification that its reception has been of a character to repay the authors for their painstaking effort to produce a book which nurses of average intelligence can readily understand, and, if they have the inclination, can also turn to good account in the pursuit of their avocation.

A Manual of Fever Nursing. (London: Rebman.)

The "Manual of Fever Nursing," by Dr. R. W. Wilcox, a review of which appeared in our columns last October, is now published by Messrs. Kegan Paul, Trench, Trübner, and Co., Limited. The price is 4s. 6d. net.

The Englishwoman's Year Book and Directory, 1905. (London: Adam and Charles Black. 2s. 6d.)

The "Englishwoman's Year Book" has now lived for a quarter of a century, and we judge by the annual edition just issued that it will attain its centenary. We have no hesitation in saying that no girl who has to earn her living in the world or who desires to enter the field of women workers can afford to be without this book. Miss Emily James, the editor, takes care to add to its value year by year. Thus the present volume contains new articles on elementary school teaching, education in India, laundry work, journalism for women, elocution work, and secretarial work. The information in its pages respecting the numerous professions and branches of industry open to women is, of course, all the more useful because it is supplemented by a list of the names and addresses of officials to whom inquiries should be made.

### TRAVEL NOTES AND QUERIES.

By our Travel Correspondent.

Venice for Three Days (A Countrywoman).—I shall be pleased to help you, for I like your spirit and pluck in making the best of the limited time and money at your disposal. I feared the journey would be too much, but I can tell you of one or two places new to you where living is very cheap. Write me fully how long your holiday will be and exactly how much you can spend. I think you may enjoy a foreign holiday quite within your means. Letters are only seen by me, so that you can write quite frankly. Belgium is the cheapest country, and next comes Brittany, where living is very cheap, but the journey is more expensive.

Bruges or Paris (Old Maid).—I think you would do well to choose Bruges. Paris is not pleasant in July; it is the off season, and often very hot and glaring, and sometimes a little smelly. Moreover, it is dear, and you would spend as much or more for a week in Paris as for a fortnight in Bruges. (1) Expense of journey from London to Bruges and back, *via* Tower Bridge and Ostend, second-class return, about 12s. If you do not like to go all the long way by sea, the second return *via* Dover and Ostend is £2. At Bruges accommodation can be had cheaply, from 6 francs, which is 5s. per day; roughly speaking, £3 12s. for 15 days. Then there will be tips in hotels, coming to 5s. more, or perhaps 6s. or 7s., according to circumstances. At the highest figure your hotel expenses would, therefore, be £3 19s. each for the fortnight. Then for excursions—that question depends entirely upon what you can afford to spend. You need spend very little, as there is plenty to see close at hand, but you may spend much, as all the finest of the Flemish cities are within a day's excursion. (2) Second-class return ticket to Paris, *via* Newhaven and Dieppe (night service), £2 2s. 3d. You must, I think, cross by night, because you would be unable to reach London in time from the Midlands. The cheapest accommodation I could recommend you in Paris would be 7s. per day. Seven days would be £2 9s. Tips not less than 8s. for the week. This, you see, makes £2 17s. Then museums, galleries, and omnibuses will mount up. I should advise Bruges and not Paris most distinctly. Write me again as to your decision and I will advise further and give you addresses.

Torquay or North Cornwall (Microbe).—Torquay is very expensive, and I think, on the whole, Ilfracombe would suit you better. It is not quite so dear and is, on the whole, a brighter place, and there are numerous delightful excursions to be made. If you prefer Torquay, write to the following addresses and ask their Pension terms:—Clarence Hotel, Petworth Private Hotel, Chestnut Avenue, St. James' Hotel. Mr. Honeycombe, proprietor, takes guests from £2 2s. per week. At Ilfracombe write for terms to Mr. Myatt, "Ilfracombe Grand Private Hotel," Capstone Parade. He offers terms from 1½ guineas. Also to Mr. Price, "Capstone Boarding House." Also to Mr. Brokenbrow, "The Belgrave Hotel." I gather from your letter that you want a lively place and I think Ilfracombe is the spot for you. From there you can take numerous coach excursions and see a great deal. Cornwall is too quiet for you and too much like your present surroundings. You would do well, I think, to get Pearson's 1s. "Ilfracombe and District" of the series called "Gossipy Guides." Any bookseller will get it for you and it will supply all the information you need.

Rules in Regard to Correspondence for this Section.—All questioners must use a pseudonym for publication, but the communication must also bear the writer's own name and address as well, which will be regarded as confidential. All such communications to be addressed "Travel Correspondent, 'Nursing Section of *The Hospital*,' 28 Southampton Street, Strand." No charge will be made for inserting and answering questions in the inquiry column, and all will be answered in rotation as space permits. If an answer by letter is required, a stamped and addressed envelope must be enclosed, together with 2s. 6d., which fee will be devoted to the objects of "The Hospital" Convalescent Fund. Ten days must be allowed before an answer can be published.

### The Vaginal Douche in District Nursing.

BY A SUPERINTENDENT OF DISTRICT NURSES.

In many complications of childbirth the treatment usually ordered by the doctor is the vaginal douche, and terrible are the variations of this treatment as carried out by the well-meaning but uninstructed mothers, aunts, sisters, and friends. The patient is generally drawn to the edge of the bed and all clothing is removed. The water is expected to find its way into a basin stood on the floor. The syringe is thrown uncarbolised on to some table or chair, and left there until required again. The temperature of the douche is never taken, and the first is usually too cold, and the subsequent ones too hot.

The following is a typical case where vaginal douches were required, and full details are given as to the best way of administering them in district work.

The patient was the wife of a soldier, and was shortly expecting her confinement. As she was not on the strength of the regiment she was living outside the barracks in small lodgings. She was an extremely refined and intelligent woman, and had lived in good service until her marriage with the handsome young soldier, who had, unhappily, what she discreetly described as "an unquenchable spark in his throat." To keep the wolf from the door the poor woman had taken in officers' washing, and the work had been too much for her. We went one evening into her little back kitchen about a month before the baby was expected, and found her suffering such intense pain that we felt no doubt that labour had already commenced. Unfortunately her alleged "stand-offishness" made her anything but a favourite with her neighbours, and while humanity plainly demanded that she should not be left for a moment alone, no one would remain with her, and although it is against the rules of our local association we decided that we must do so ourselves. We sent the boy-husband to tell the doctor and beg him to come as soon as he could, and then persuaded the woman to come upstairs. We lighted a nice little fire and prepared the bed. On the right hand side of the bed we placed a thick sack, a blanket, three layers of stout brown paper and a clean sheet, and on the left hand side another sack and a clean piece of sheeting. We dressed the patient in a clean chemise and nightdress, pinning them up high under the arm with safety-pins, and putting on a warm shawl. We took glycerine and perchloride out of our bag, and by that time the doctor had arrived. He made a careful examination, said that the event could not occur for at least two hours, and that he would go home to dinner and return again. While he was gone we were able to get everything ready. We cleared the dressing-table, covered it with clean paper, and arranged all that was necessary to give a vaginal douche.

We placed by the fire:—(1) A clean chemise; (2) a draw sheet; (3) a diaper; (4) a binder.

On the table:—(1) A small piece of mackintosh; (2) four small pieces of wool and some pieces of soft linen to be dipped in antiseptic lotion and wrapped round the left hand; (3) a quart jug for lotion; (4) a Higginson's syringe with glass nozzle; (5) soap dish with lotion at right hand corner of mackintosh; (6) receiver at left-hand corner; (7) three sets of strands of thread each containing six strands and knotted at either end; (8) a blunt-pointed pair of scissors; (9) a little Condy's fluid in a basin (we were three miles from the Home and could get nothing else).

We then arranged all the clothes and requisites for the baby, but as there was very strong reason to believe it would be born dead, we sent the husband secretly to the grocer's to get a neat wooden box with a lid, and a sheet of white wadding.

We placed an empty bucket outside the bedroom door, and a clean vessel with water in it to receive the after-birth. We drew a jug of cold water, and placed a kettle of water on the fire to boil.

Our preparations being now complete, we gave the patient a little refreshment, and devoted ourselves to trying to keep up her courage. The doctor soon returned from his dinner, and seconded our efforts by telling the patient amusing stories, and at eleven o'clock the baby was born. It had been dead for some days. We received it in the shawl, and laid it on one side while we gave the douche. The first douche must always be given on the side. We then bathed her, changed the body linen, put on diaper and binder, and moved her to the clean dry side of the bed.

We washed the infant's body, and cut the wadding into a shroud, and asked the husband to take the little coffin to the cemetery on the following day and give it to the sexton. It is always advisable for the nurse to make these arrangements in very poor homes, as otherwise the most brutal callousness may be shown in the method of disposing of the body.

The doctor asked us to undertake the further treatment of the case, and to give two vaginal douches daily.

To prepare the douche, have ready—(1) Kettle of boiling water; (2) jug of cold water; (3) chamber; (4) mackintosh, or substitute; (5) draw-sheet; (6) diaper and binder.

Cover a table with clean paper and arrange as previously described. The douche must then be prepared, whether carbolic, Condy, boracic, or iodine. In this case the doctor ordered boracic, two teaspoonfuls to the quart, and the temperature when applied was to be 100° F. We therefore made it 106° F., as several degrees are inevitably lost in drawing the water through the syringe.

Prepare the bed and put one blanket double over the chest and one double over the knees, thus making it possible to apply the syringe with the least practicable exposure of the patient. Put a pillow under the lower part of the back to support the hips, and then slip in the sacking and draw-sheet. The nurse should then take off binder and napkin, and carbolise and wash her hands. After washing between the labia and putting the chamber in place, she must again wash her hands and disinfect them. Insert the nozzle of the syringe and gently press about 18 times, being careful that no air is injected with the fluid. Withdraw the diaper, wipe patient well with a warm cloth, take away chamber and draw-sheet. Put on clean diaper and binder, and, the patient being now comfortable, proceed to clear away in fixed order:—(1) Burn dirty dressings. (2) Carbolise the Higginson's syringe. This should be done before and after use. Then wrap it lengthwise in a clean towel and hang it in a cupboard, well out of reach. Put the glass nozzle into a glass jar with carbolic, cover it with paper to keep out the dust, and affix a poison label. (3) Throw away the douche and notice the nature of the discharge. Throw away the rest of the lotion. (4) Return all bottles, etc., to the bag and put all articles used for the douche together in one place. (5) The nurse should again wash her hands, and then write report for the doctor.

Fixed order and method is the only way in which rapidity and efficiency can be combined, and it is not too much to say that in some cases there can be no efficiency without rapidity. By rapidity is of course meant a method of action as far removed from hurry as the strong, swift flight of an eagle from the aimless, frightened flutterings of a barn-door fowl.

If the friends can possibly afford it they should buy a syringe or a douche-can to be kept for the exclusive use of the patient.

### A Book and its Story.

AN EIGHTEENTH CENTURY ROMANCE.*

The story of Nancy Stair, the only child of the marriage in 1768 of Lord Stair, to the lovely half-gipsy girl, Marian Ingarrach, who died a year later, is told by the author in a simply charming way. No more romantic situation would be needed for the basis of a novel whose characters were invented to make a sensational piece of fiction, but here the characters are real, and the incidents, those which are more or less a matter of history. Humour and pathos mingle in a natural, human fashion, and give the finishing touch to the most charming book, of its kind, that has appeared for some time.

Lord Stair's wooing of his bride was of the briefest. He met her on a cruise round the west coast of Scotland in search of any adventures that might turn up. So far he had not responded to the advances of the dowagers of the day with daughters to dispose of, and had come away heart-whole, with a party of men friends, seeking immunity from their attentions. One June night Lord Stair and his friends put in under the lee of the shore, and made their way up the cliff to a little inn whose lights were the only visible sign of a habitation. While Lord Stair is waiting in the little parlour for the men who have gone to inspect the accommodation offered, the sound of movement arrests his attention, and he has the first view of his fate. "As I stood I heard a sudden noise, saw a curtain of a door at the side raised, and a girl in a black robe, with a lighted candle in her hand, looked in at me.

"For twenty-seven years I had waited for a sight of that girl.

"I do not know how love comes to other natures than my own (but tales are not wanting of the men of Stair who had lost their wits when crossed in love), and men of notable integrity have told me how leisurely they strolled into the condition of loving; but for me, by one questioning glance from a pair of eyes, half grey, half blue, I was sunk fathoms deep in love, in love that knows nothing, cares for nothing but the one beloved. Soul and body I was signed, sealed, and delivered 'hers' in that first sight I had of her in the doorway with the candle in her hand and the crimson curtain framing her as if [illegible] a picture. . . . At the first gaze with each other we stood, no word spoken between us, for full a minute of time, when the noise of the men coming back disturbed her; she dropped the curtain, and the light of her candle disappeared, a little at a time, as though she were walking from me down some long passage-way."

The attraction was mutual, and Lord Stair, true to family traditions, lost no time in making the lovely girl his own. A few days later, "Father Pierre," we read, "made Marian and myself one in marriage, such as the gods intended when the world was young and the age of the world gold." One brief year of happiness spent in the little inn bought by Lord Stair from the old hostess who had reared his wife as her own child, and then, heartbroken and despairing, he is left a widower, with the little girl Nancy as the link between a past all too transient. Years after he writes of this period with pathetic insistence, and recalls the picture of those early days of married life: "And the time is all of Marian; Marian standing in white in the going down of the brae-side to welcome me; Marian on my knee in the twilight, looking out seaward and starward; Marian with her brown head, and face such as the angels have, resting on my breast in the dawning. Marian! Marian! Marian! I, an old man, who was once that bonny Jock Stair, all your own, call to you. Can you come? Will it ever be again? See I stretch my hands, wrinkled, old, to that far-off blue, and ask you, as I have a thousand times, to send me peace."

The shock of his wife's death brings on a serious illness, and after four months, when Lord Stair is convalescent, he takes the tour round the world which was customary towards the end of the eighteenth century. It was five years before he came home again, and the first news he had of his child is from his old friend Sandy Carmichael, who reminds him that she needs him now that she is growing a big girl. "'And what of my girl?' I asked. 'Nancy,' he said, a curious look coming into his face as he smiled, 'she's one you must see to judge of for yourself. I've raised her up as well as I could. I've spent time with her!'"

Nancy has been left in charge of Dame Dickenson, who had been foster-parent to her own mother. Lord Stair detects in the reticence of Sandy Carmichael's reply to his inquiries about Nancy, that she is not quite an ordinary child. He had not long to wait for the realisation of this impression, for in answer to a written request to Dame Dickenson to bring Nancy at once to Stair Castle, he receives a large sheet of paper folded in a curious fashion, having no address on it, upon which, in an irregular childish hand, not even at this date without character, the following words were written:

"I am not comming.
"Nancy Stair."

So Lord Stair, wondering what kind of rebellious daughter this was who at such an early age defied her father's wishes, had to ride over to Landgore and make her acquaintance himself. This is the tender reminder of a vanished past that stood before him as he rode up.

"She wore a short-waisted black frock, with a very long skirt, which almost touched the ground. On her feet were red shoes, which twinkled in and out the black, as with great dexterity and lightness she clambered up the steps of the porch and stood before me, one of the miracles of God before which we human folk stand abashed. For here was Marian again! Marian to the turn of an eyelash—in the bronze chestnut curls which stood like a halo round her face . . . . in all the dear, quaint, beautiful baby devouring me with grey eyes, and looking at me with a shy, radiant smile, as she held a kitten tail up against her breast. 'Jock,' she cried with a rapturous smile."

If the story of Nancy extended no further than her childhood it would in itself be one of unusual attraction, and the following interview between her father the next day proves the sweet thoughtfulness and sense of justice to those who, even at this early stage, were her willing slaves and companions. Can any picture be quainter than the following? Lord Stair calls to her as she stands in the garden surrounded by her live stock: "Hello, Little Flower, are you willing to come to Stair with me?" "We're getting ready, Jock," she answered, putting her hand in mine. "We," I inquired. "Whom do you mean?" "Nancy Stair," she answered, touching herself on the breast with a forefinger, "Dame Dickenson, Father Michel, the two or three dogs, the kittens, the one without a name, the drey hen, and the broken owl." "Nancy Stair," I broke in with firmness, "it will be utterly impossible to take all these folk up to Stair Castle." . . . . "I can't go, Jock, I can't go." "Why?" I inquired. "Oh! don't you see? I can't go and leave all my people, Jock?" From childhood to womanhood, gifted, beautiful, tender above all her contemporaries, one follows the story of Mistress Stair through the pages of Mrs. Lane's charming romance with an interest that never flags. We hope it may be the good fortune of our readers to follow it also and experience the same pleasure in its perusal that we have found.

* Nancy Stair. By Ellinor M. Lane. W. Heinemann. 6s.

### Notes and Queries.

**REGULATIONS.**

**The Editor is always willing to answer in this column, without any fee, all reasonable questions, as soon as possible.**

**But the following rules must be carefully observed.**

1. **Every communication must be accompanied by the name and address of the writer.**
2. **The question must always bear upon nursing, directly or indirectly.**

**If an answer is required by letter a fee of half-a-crown must be enclosed with the note containing the inquiry.**

*Brougham Ambulance.*

(1) Can you give me the address of the firm (I believe in Lancashire) who are advertising a brougham ambulance?—*Japan.*

Messrs. Wilson and Stockall, ambulance specialists, Bury, Lancashire.

*Duties of Wardmaids.*

(2) I should be obliged if you would be good enough to inform me of the duties of wardmaids in workhouse hospitals.—*Trained Nurse.*

They vary, and, excepting duties which are obvious, each matron and head nurse decides for herself.

*Institutions for Consumptives.*

(3) Are there any institutions in this country for the free treatment of consumptives?—*Nurse.*

See the list of sanatoria published by the Society for the Prevention of Tuberculosis, price 7d., post free, obtainable from the Scientific Press, 28 & 29 Southampton Street, Strand, London, W.C.

*Nursing Home.*

(4) Can you please tell me the number of Miss ——'s Nursing Home, Wimpole Street?—*Sister W.*

Wimpole Street will be quite sufficient.

*Children's Nurses.*

(5) Kindly give me the addresses of any institutions in the British Isles where young women can be trained as children's nurses for private families other than the Norland Institute, London?—*Nurse A. R. P.*

Liverpool Ladies' Sanitary Association, 8 Sandon Terrace, Liverpool; Princess Christian Training College, Withington, Manchester; Gloucestershire School of Domestic Science, Belmont House, Cheltenham.

*District Nursing.*

(6) I am anxious to begin district nursing on my own account among patients who are able to pay a fee. Will you kindly tell me what charges to make and the best way to set to work to become known?—*Nurse A.*

The usual charge is about 2s. 6d. for an hour or part; 12s. 6d. for one daily visit and 18s. 6d. for two daily visits per week, and 5s. for an emergency visit. But fees must depend partly upon your district. Make yourself known to the medical men in your neighbourhood and by advertising.

*Midwifery.*

(7) Please can you tell me if there is any hospital which trains probationers for midwifery without premium?—*A. H.*

Write to the Secretary, Rural Midwives Association, 47 Victoria Street, S.W.

*Nurses' Clubs.*

(8) I shall be much obliged if you can tell me of a nurses' hotel or club in town where one would be received for a night or for a week end. I had an unpleasant experience in town the other day. . . .—*Country Cousin.*

The Nurses' Hostel, Francis Street, Tottenham Court Road, or St. Andrew's House Club, Mortimer Street, W.

*Training.*

(9) Can you please tell me of any institution or hospital where they teach probationers free. I do not mind if there is no salary?—*N. H.*

Most of the hospitals train their probationers free. See "The Nursing Profession: How and Where to Train."

**Handbooks for Nurses.**

| | Post Free. |
|---|---|
| "How to Become a Nurse: How and Where to Train." | 2s. 4d. |
| "The Nurses' Dictionary" (Pronouncing) ... ... | 2s. 0d. |
| "Nursing: its Theory and Practice" (Lewis.) ... ... | 3s. 6d. |
| "The Light Treatment" (just published) ... ... | 2s. 6d. |
| "A Complete Handbook of Midwifery." (Watson.) ... | 6s. 4d. |

Of all booksellers or of the Scientific Press, Limited, 28 & 29 Southampton Street, Strand, London, W.C.

### For Reading to the Sick.

"UNTO THEE I LIFT MINE EYES."

Darkness and light are both alike to Thee;
 Therefore to Thee I lift my darkened face;
Upward I look with eyes that fail to see,
 Athirst for future light and present grace.
I trust the hand of Love I scarcely trace.
 With breath that fails I cry, Remember me;
Add breath to breath so I may run my race
 That where Thou art there may Thy servant be
For Thou art the gulf and fountain of my love,
 I unreturning torrent to Thy sea,
Yea, Thou the measureless ocean for my rill:
 Seeking I find, and finding, seek Thee still:
And oh! that I had wings as hath a dove,
 Then would I flee away to rest with Thee.

*C. Rossetti.*

It is common to suppose that a passive acquiescence in the trials of life is the fulfilment of their intended end. On the contrary, it is their least important result. The real gain to be obtained through the discipline of God in any appointed trial depends on an active co-operation with it. The will must be conformed as well as subdued. It must unite with God, not merely yield to God. A true living union rests on co-operation, not on submission. To infuse into the soul in every trial the lesson it would teach, the spirit of sacrifice it involves, or the self-denial it would elicit, the secret conquest of temper or the increasing earnestness of faith, the sweetness of patience or the largeness of love, which it is intended to mature, as the needed advance in the progressive development of the soul's life—is the result contemplated

*T. T. Carter.*

Thou wert always our Father! Each sun that arose
Has done nothing through life but fresh mercies disclose;
But we feel, while the joy of our life is laid low,
Thou has ne'er been so tender a father as now.

*Faber.*

Is it not the experience of all of us? Year after year God has poured out His loving-kindness upon us. What if He had been rough, and hard, and impatient with us? What if He had cut us off in our early sins? It is God's long-suffering gentleness which is so wonderful; He bears with us so long and so unweariedly; it is always His method. With the thoughts checked, and the lips guarded, and the will set on the imitation of Jesus Christ in His Passion, we can go forth with fresh courage to meet the provocations of life, and gradually—perhaps very gradually—we shall learn to be patient, as we learn that to be patient is to be like God.—*Randolph.*

Thy work this hour is Patience! If the Past
 Hath set its image there where naught decays,
Deny not its own work to this thy last.
 Stray yearnings ever mark'd thy vanished days
 And outstretch'd longings after absent ways;
That all is past; and now thy heart incline
 To seize the present good as by it strays!
To Heaven's all-gracious Will thyself resign!
The Heavenly Kingdom this; and this is Life Divine.

*Williams.*

